# Influence of idiopathic epilepsy on blood pressure and electrocardiography in dogs treated with phenobarbital

**DOI:** 10.14202/vetworld.2024.356-360

**Published:** 2024-02-15

**Authors:** Yolanda Paim Arruda Trevisan, Maria Sabrina de Freitas, Maria Natalia de Freitas, Jaqueline Konrad, Juliano Bortolini, Ana Carolina Schipiura, Ana Flávia Borges de Freitas, Sarah Szimanski Pazzini, Arleana do Bom Parto Ferreira de Almeida, Valéria Régia Franco Sousa

**Affiliations:** 1 Department of Internal Medicine for Small Animals at the Veterinary Hospital of the Federal University of Mato Grosso. Av. Fernando Corrêa da Costa, nº 2367, Bairro Boa Esperança, Cuiabá, Mato Grosso, CEP: 78060-900, Brazil; 2 Department of Statistics of the Federal University of Mato Grosso. Av. Fernando Corrêa da Costa, nº 2367, Bairro Boa Esperança, Cuiabá, Mato Grosso, CEP: 78060-900, Brazil; 3 Department of Clinical Pathology Laboratory at the Veterinary Hospital of the Federal University of Mato Grosso. Av. Fernando Corrêa da Costa, nº 2367, Bairro Boa Esperança, Cuiabá, Mato Grosso, CEP: 78060-900, Brazil

**Keywords:** arterial hypertension, cardiac conduction, electrocardiogram, epileptic seizures, hemogasometry, tachycardia

## Abstract

**Background and Aim:**

Dogs with idiopathic epilepsy (IE) experience a shortened lifespan, neurobehavioral changes, and an increased risk of comorbidities during the interictal period. There have been several reports of sudden death in humans with epilepsy, suggesting changes in cardiac rhythm secondary to seizures. In veterinary medicine, there are still no such conclusive studies. The present study aimed to evaluate blood pressure values, electrocardiographic findings, and laboratory parameters in dogs with IE treated with phenobarbital and to correlate these findings with possible cardiac alterations.

**Materials and Methods:**

Twenty-one dogs were divided into 11 healthy dogs and 10 idiopathic epileptic dogs for blood analysis, computerized electrocardiogram, and oscillometer-based blood pressure measurement.

**Results:**

QRS complex and S-T interval values differed significantly between groups, but blood pressure values were not significantly different.

**Conclusion:**

IE can occur with alterations in cardiac conduction and is a pathological condition.

## Introduction

Idiopathic epilepsy (IE) is a chronic neurological disease with a prevalence of 0.75% in dogs, which is similar to the prevalence of epilepsy in humans. IE is associated with reduced life expectancy, neurobehavioral changes, and an increased risk of comorbidities during the interictal period [1, 2]. IE is diagnosed by exclusion because affected dogs show no clinical, laboratory, or imaging alterations [3]. There have been several reports of sudden death in humans with epilepsy [4–6]; however, the mechanisms underlying these deaths remain unclear, even though arterial hypertension has been shown to lower seizure threshold and precipitate epileptic seizures. Seizures are known to be related to an increase in sympathetic tonus, which, in turn, increases blood pressure [4].

In addition, increased sympathetic stimulation leads to catecholamine toxicity in the myocardium, which promotes acute cardiomyopathy. Catecholamine toxicity and low sensitivity of baroreceptors may also contribute to increased blood pressure [5]. Epilepsy affects both cardiac rhythm and frequency due to autonomic neuronal dysfunction in addition to electrocardiographic changes, such as increased QT intervals and decreased RR intervals, due to increased post-seizure heart rates (HRs) [6].

Domestic dogs provide an ideal model for translational medicine because they have the most phenotypic diversity, and data related to dog health presents many opportunities to discover insights into health and disease outcomes in both populations [7]. Unlike human medicine, there is a paucity of information on the relationship between IE in dogs and hypertension or changes in heart rhythm. Therefore, the present study evaluated blood pressure values, electrocardiograph findings, and laboratory parameters in dogs treated with phenobarbital.

## Materials and Methods

### Ethical approval

This study was approved by the Ethics Committee for the use of animals under protocol number 23108.934331/2018-80.

### Study period and location

This study was conducted from March 2021 to July 2023. All dogs in this study were obtained from the Veterinary Hospital of the Federal University of Mato Grosso, Cuiabá, Mato Grosso, Brazil.

### Animals

Twenty-one dogs of different ages, breeds, and sexes were selected and divided into two groups: group one (G1) consisted of 10 dogs diagnosed with IE, with no history of seizures in the past 7 days, according to the diagnostic criteria Tier I described by De Risio *et al*. [8] with complete blood cell count, serum biochemistry profile (sodium, potassium, chloride, calcium, alanine aminotransferase, alkaline phosphatize, urea, creatinine, total protein, albumin, glucose, and lactate), and urinalysis. Group two (G2) consisted of 11 healthy dogs without changes in physical, neurological, and laboratory examinations and without epileptic seizures. To ensure standardized results, the same veterinarian performed all neurological examinations. All G1 dogs were treated with phenobarbital for at least 60 days.

### Complementary examinations

All dogs from G1 and G2 underwent the following tests: Complete blood count, serum biochemistry profile (alanine aminotransferase, alkaline phosphatase, urea, creatinine, albumin, glucose, and lactate), urinalysis (specific gravity, protein, glucose, pH, and sediment cytology), and venous blood gas analysis according to De Risio *et al*. [8] for the diagnosis of IE.

A complete blood count was performed on each dog using the Poche 100iV Automated Hematology Analyzer (Roche, Switzerland) and light microscopy (Leica Microsystems®, DM500, Wetzlar, Alemanha, Germany), serum biochemistry was performed using the Automatic Analyzer CM 250 (Wiener Lab., Argentina), lactate measurement was performed using the Accutrend Plus (Roche, Switzerland), and blood glucose measurement was performed using the Accu-Chek Active (Roche, Switzerland) [9]. Urinalysis was performed using physical assessment, refractometry, Combur-Test reagent strips (Roche, Switzerland), and sedimentoscopy (Leica Microsystems). Venous blood gas analysis was performed using a Cobas b 121 device (Roche, Switzerland) [9]. Electrocardiography was performed through computerized interpretation of an electrocardiogram (Tecnologia Eletrônica Brasileira) using measurements obtained according to Varshney [10]. Blood pressure was obtained using petMAP (Ramsey Medical, INC. of Tampa, FL, USA) using an oscillometer method, which takes five measurements and discards the first [11].

### Statistical analysis

The data were analyzed for normality using the Shapiro–Wilk test and, subsequently, the Student’s or Welch’s t-test, with p < 0.05 indicating statistical significance using Jamovi software version 2.3 (https://www.jamovi.org/)

## Results

G1 included 10 dogs with epilepsy (G1); 6 (60%) mixed-breed dogs and 4 (40%) defined breed dogs (1 Pinscher, 1 Australian Cattle Dog, 1 Spitz, 1 Poodle), 1–7 years of age (mean = 3.40, median = 3 years), consisting of 7 (70%) males and 3 (30%) females. G2 included 11 healthy dogs; 4 (36.4%) mixed-breed and 7 (63.6%) defined breed dogs (2 Spitz, 2 Pug, 1 French bulldog, 1 Dachshund, 1 Chow Chow) (p = 0.302), 1–6 years of age (mean = 3.82, median = 4 years), consisting of 7 (63.3%) males and 4 (36.4%) females (p = 0.772).

All dogs with epilepsy were treated with phenobarbital monotherapy at the time of this study. The mean and median phenobarbital dose was 3 mg/kg, which promoted a mean and median serum concentration of 24.37 mcg/mL and 22.5 mcg/mL, respectively. G1 had a mean and median frequency of epileptic seizures of 1.8 and 2 generalized seizures in 24 h, respectively, and 30% of the dogs had satisfactory control after starting treatment.

Table-1 shows the mean, median, standard deviation, and interquartile ranges for the hematological, biochemical, and urinary analyses for the two groups. The hemoglobin, hematocrit, creatinine, and glucose values (p *=* 0.05) were significantly lower in dogs with epilepsy than in healthy dogs, although these values were still within the normal reference range. Table-2 shows blood gas analysis data, including mean, median, standard deviation, and interquartile range values. The average ionized calcium level was subtly lower, whereas the average bicarbonate level was slightly higher in the G1 dogs than the reference values, although the difference was not statistically significant.

**Table-1:** Values of statistical analyses of hematological, biochemical, and urinary findings in groups of dogs with idiopathic epilepsy and control group.

Variables (Reference)	Control group		Idiopathic epilepsy group		p-value*
	
	Mean (SD)	Median (IQR)	Mean (SD)	Median (IQR)	
Hematocrit (37–55%)	55.64 (4.59)	57.00 (6.50)	46.24 (4.83)	46.00 (6.20)	<0.001
Hemoglobin (12.0–18.0 g/L)	18.14 (1.63)	17.70 (2.60)	15.72 (1.71)	15.70 (2.28)	0.004
Total leucocytes (6.0–17.0 mil/mm^3^)	9.82 (2.70)	9.10 (1.05)	11.66 (3.34)	10.85 (4.00)	0.179
Platelets (200–500 mil/mm^3^)	331.91 (94.19)	330.00 (189.00)	281.10 (154.44)	249.00 (225.75)	0.369
Total plasma proteins (6.0–8.0 g/dL)	7.44 (0.56)	7.40 (0.70)	7.80 (1.0)	7.70 (1.25)	0.311
Urea (21–59.9 mg/dL)	32.10 (7.14)	31.00 (4.75)	33.10 (12.55)	32.50 (11.0)	0.829
Creatinine (0.5–1.5 mg/dL)	1.12 (0.19)	1.05 (0.18)	0.91 (0.20)	0.90 (0.28)	0.029
Albumin (2.6–3.3 mg/dL)	3.27 (0.32)	3.30 (0.40)	3.47 (0.43)	3.50 (0.83)	0.248
Alanine Aminotransferase (21–102 U/L)	46.55 (19.98)	43.00 (25.50)	77.00 (79.34)	40.00 (38.50)	0.265
Alkaline phosphatase (20–156 U/L)	72.36 (28.29)	85.00 (22.50)	194.70 (231.79)	83.00 (57.50)	0.131
Glucose (65–118 mg/dL)	77.55 (13.14)	80.00 (20.00)	91.00 (3.03)	91.50 (4.00)	0.007
Lactate (0.3–3.2 mmol/L)	3.37 (0.96)	3.30 (1.15)	3.09 (0.67)	2.80 (0.60)	0.502
Urinary pH (5.5–7.5)	6.44 (1.24)	7.00 (2.00)	7.00 (1.31)	7.00 (2.0)	0.382
Urinary Density (1.015–1.045)	1.030 (11.11)	1.030 (13.75)	1.027.38 (10.54)	1.028.00 (20.00)	0.578

p value column presents the result of t or Welch test . SD=Standard deviation, IQR=Interquartile range

**Table-2:** Values of statistical analyses of venous blood gas in groups of dogs with idiopathic epilepsy and control group.

Variables (Reference)	Control group		Idiopathic epilepsy group		p-value
	
	Mean (SD)	Median (IQR)	Mean (SD)	Median (IQR)	
pH (7.31–7.42)	7.42 (0.05)	7.40 (0.02)	7.40 (0.10)	7.38 (0.07)	0.170
Bicarbonate (17–24 mEq/L)	20.69 (3.37)	21.1 (2.85)	22.90 (2.83)	22.39 (2.41)	0.248
Sodium (140–155 mmol/L)	147.27 (2.85)	147.35 (4.37)	151.50 (4.40)	150.94 (3.28)	0.096
Potassium (3.7–5.8 mmol/L)	3.97 (0.17)	3.99 (0.16)	4.17 (0.59)	4.14 (0.62)	0.488
Ionic calcium (1.2–1.5 mmol/L)	1.29 (0.07)	1.29 (0.05)	1.30 (0.56)	1.11 (0.33)	0.166
Chlorides (105–120 mmol/L)	110.29 (3.03)	110.7 (3.05)	112.15 (5.95)	113.78 (5.37)	0.103
Base Excess ecf* (−3–+3)	−3.61 (2.96)	−3.35 (2.40)	−2.10 (1.88)	−2.58 (2.21)	0.424

p value column presents the result of t or Welch test. SD=Standard deviation, IQR=Interquartile range.

* ecf=Extracellu lar fluid compartment.

Table-3 shows the electrocardiographic and blood pressure measurements. The QRS complex and S-T interval values significantly differed between G1 and G2, with the mean values of G1 being higher than that of G2. Diastolic blood pressure, systolic blood pressure (SBP), and mean blood pressure values in G1 were higher than those in G2, although the differences were not statistically significant.

**Table-3:** Values of statistical analyses of electrocardiogram and blood pressure in groups of dogs with idiopathic epilepsy and control group.

Variables (Reference)	Control group	Idiopathic epilepsy group			p-value
	
	Mean (SD)	Median (IQR)	Mean (SD)	Median (IQR)	
p wave duration (20 a 40 ms)	45.9 (10.71)	47 (5.5)	53.88 (6.64)	53.00 (5.25)	0.082
p wave amplitude (0.15–0.40 mV)	0.22 (0.075)	0.23 (0.08)	0.17 (0.05)	0.17 (0.08)	0.106
P-R interval (80–120 ms)	88.54 (13.29)	92 (13.29)	91.25 (22.46)	87.50 (20.50)	0.746
QRS complex (30–50 ms)	48.45 (12.04)	42 (12.04)	60.13 (9.58)	57 (9.50)	0.037
R wave amplitude (0.9–2.4 mV)	0.84 (0.29)	0.85 (0.38)	1.19 (0.48)	1.10 (0.53)	0.070
ST interval (40–100 ms)	107.27 (45.91)	127 (72.5)	153.13 (14.48)	152.00 (21.75)	0.009
QT interval (110–230 ms)	175.27 (44.38)	187 (36.5)	208.75 (15.31)	203.50 (21.25)	0.058
HR (60–150 bpm)	146.90 (20.88)	144.00 (13.00)	129.19 (32.77)	132 (23.38)	0.146
DBP (70–90 mmHg)	95.55 (9.98)	97.00 (5.75)	102.89 (21.69)	104.50 (24.63)	0.320
MBP (90–100 mmHg)	122.98 (14.10)	122.00 (9.25)	138.91 (30.75)	146.50 (40.00)	0.141
SBP (140–160 mmHg)	176.36 (25.72)	174.75 (30.50)	204.72 (43.90)	217.25 (43.00)	0.079

p value column presents the result of t or Welch test. SD=Standard deviation, IQR=Interquartile range, HR=Heart rate, DBP=Diastolic blood pressure, MBP=Mean blood pressure, SBP=Systolic blood pressure

## Discussion

Dogs with epilepsy had a history of seizure onset between 6 months and 6 years of age, but no history of status epilepticus. In the present study, monotherapy with phenobarbital was the first-line treatment to control epileptic seizures; however, it is common to include other drugs (s) to improve the control [12]. Continuous use of phenobarbital can result in anemia induced by a decrease in the production of erythrocytes in the bone marrow, the destruction of erythrocytes by immune-mediated processes, and an increase in hemoglobin degradation [13]. Data from the present study showed lower hemoglobin and hematocrit levels (p < 0.05) in dogs with epilepsy compared with healthy control dogs, suggesting that pharmacological treatment for epilepsy may have influenced these results.

There were no significant changes in other hematological and urinary parameters; however, liver enzymes should be carefully evaluated in dogs treated with phenobarbital for epilepsy because drug-induced liver disease may occur. As shown in Table-1, alkaline phosphatase values were higher in G1 dogs than in G2 dogs, although the differences were not statistically significant, reinforcing the idea that phenobarbital is metabolized by cytochrome P450 enzymes, leading to mitochondrial dysfunction and oxidative stress [13]. Although the serum creatinine and glucose concentrations of dogs with epilepsy were within the reference ranges, they differed significantly from those of healthy dogs, which may result from metabolic changes caused by the continuous use of phenobarbital [14].

The mean ionic calcium level was below the reference range in G1 dogs, which may result in a prolonged QT interval. The diastolic, mean, and SBPs were increased in both groups, although the increase was more pronounced in the dogs with epilepsy. Epilepsy and hypertension have been observed to have a bidirectional relationship, in which brain damage resulting from increased blood pressure can decrease seizure thresholds, increasing the incidence of epilepsy in hypertensive patients [5]. Fluctuations in blood pressure related to stressful situations, such as “white coat hypertension” syndrome in humans [15], may explain the increase in blood pressure in both groups.

Although HR was not found to change in the present study, other studies evaluating cardiac changes in experimental rat models with induced epileptic seizures observed an increase in HR during the interictal period, regardless of the etiology of the seizure. However, this increase in HR may be due to poor autonomic regulation, leading to a predisposition to cardiac alterations [6].

The mean duration of the P wave on the electrocardiogram, which translates to the period of depolarization and contraction of the atrial myocardium, was longer in both groups, although this difference was not significant. A prolonged P wave indicates a delay in atrial myocardium and/or its bundle and/or atrioventricular node conduction. In patients with epilepsy, repeated autonomic activations that occur during seizures can injure myocytes and cardiac structures that conduct electrical impulses [16]. In addition, apoptosis and fibrous tissue deposition may occur in the heart muscle, leading to a delay in conduction time and, consequently, an increase in the duration of the P wave and QRS complex [17].

The duration of QRS complex is longer in dogs with epilepsy (G1), which is suggestive of left ventricular enlargement and/or a left bundle branch block. All dogs included in the present study showed an increase in the S-T interval; however, the difference was statistically greater in G1 than in healthy control dogs [10]. The prevalence of electrocardiogram abnormalities, including S-T interval changes, was higher in the epileptic group, similar to that observed in this study [18].

## Limitation

A limitation of this study is that we did not address epileptic patients who did not start treatment with antiepileptic drugs. Therefore, we did not compare electrocardiogram abnormalities between patients who used and those who did not use it. Thus, we understand the limitations of this study. Preliminary analyses suggest new pathophysiological perspectives that may be involved in epilepsy in dogs and humans and require better understanding.

## Conclusion

On the basis of the aforementioned results, it can be suggested that IE influences electrical conduction because repeated autonomic activations that occur during seizures can injure myocytes and cardiac structures. Therefore, conducting follow-up studies in different groups is important to characterize cardiac changes better. In addition, dogs with IE exhibited mild hematological changes, which can be attributed to the use of phenobarbital monotherapy, and the clinical follow-up of all patients increased.

It should be stressed that epileptic dogs must be monitored by cardiology throughout their lives due to cardiovascular complications secondary to seizures that may occur.

The prognostic significance of electrocardiography remains uncertain and longitudinal follow-up is required to understand better the progression of heart disease and its correlation with epilepsy. Because this study was cross-sectional, the absence of previous echocardiographic analysis limited a more robust analysis of relevant electrocardiographic alterations.

## Authors’ Contributions

MSDF, MNDF, and JK: Data curation. JB: Formal analysis. ACS, AFBDF, and SSP: Methodology. YPAT: Project administration; writing - original draft. VRFS and ADBPFDA: Supervision and writing – review and editing. All authors have read, reviewed, and approved the final manuscript.
